# Heterogeneity of regulatory B cells in autoimmune diseases: implications for immune equilibrium and therapeutic strategies

**DOI:** 10.1093/immadv/ltaf020

**Published:** 2025-05-08

**Authors:** Haozhen Yan, Jing He, Xiang Lin

**Affiliations:** School of Chinese Medicine, The University of Hong Kong, Hong Kong SAR, China; Department of Rheumatology and Immunology, Peking University People’s Hospital, Beijing, China; School of Chinese Medicine, The University of Hong Kong, Hong Kong SAR, China; State Key Laboratory of Pharmaceutical Biotechnology, The University of Hong Kong, Hong Kong SAR, China; Department of Chinese Medicine, The University of Hong Kong-Shenzhen Hospital (HKU-SZH), Shenzhen, China

**Keywords:** regulatory B cells, Bregs dysfunction, autoimmunity, autoimmune diseases

## Abstract

Regulatory B cells (Bregs) play a crucial role in maintaining immune tolerance and preventing autoimmune diseases. However, in autoimmune conditions, the quantity and function of Bregs are often impaired, leading to pro-inflammatory microenvironment and immune dysregulation. This review provides an in-depth examination of how Bregs are affected in various autoimmune diseases, including multiple sclerosis, rheumatoid arthritis, systemic lupus erythematosus, Sjögren’s disease, autoimmune diabetes, and other autoimmune conditions. By summarizing the alterations in Bregs phenotype and function in these specific diseases, we conclude that the Bregs response is complex and variable, showing inconsistent trend across different diseases or even within the same disease. Thus, understanding the heterogeneous nature of Bregs in the autoimmune pathogenesis facilitates novel therapeutic strategies to re-establish immune equilibrium.

## Graphical Abstract

**Graphical Abstract:**
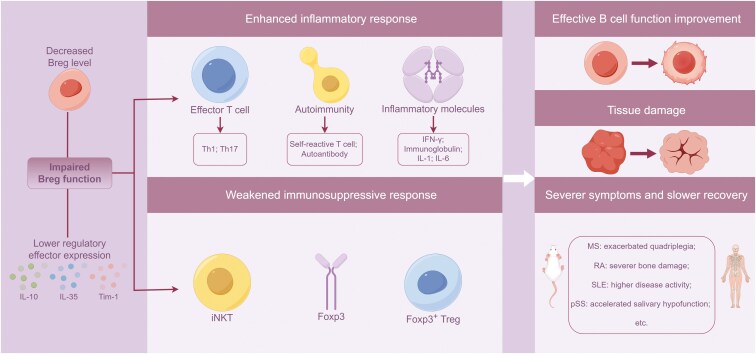

## Introduction

Regulatory B cells (Bregs) have emerged as critical modulators of immune responses, playing pivotal roles in maintaining immune homeostasis and preventing excessive inflammation and autoimmunity. Initially recognized for their antibody-producing capabilities, B cells are now understood to possess diverse regulatory functions that contribute to the suppression of both innate and adaptive immune responses [1]. These functions are primarily mediated through the production of anti-inflammatory cytokines such as IL-10, TGF-β, and IL-35, as well as through direct cell–cell interactions [2]. Notably, the plasticity and heterogeneity of Bregs underscore the complexity of their regulatory roles within different immunological contexts, e.g. autoimmune disorders.

This review aims to provide an overview of the current knowledge on Bregs, focusing on their altered phenotypes and functionality during autoimmune progression. We will also explore the potential of targeting Bregs for therapeutic interventions. Understanding the defects of Bregs in immune regulation holds promise for the development of novel strategies to modulate immune responses and promoting tolerance in clinical settings.

## History and overview of Bregs

B cells exhibiting immunosuppressive functions were initially identified in guinea pigs in 1970s [3]. Named ‘regulatory B cells’ [4], these cells were confirmed for the first time to exist in mice in 1983 [5] and in humans in 2009 [6]. Increasing numbers of studies have subsequently revealed the existence and characteristics of Bregs in mice [7] and humans [8] with autoimmune diseases (ADs).

Bregs have been found to play a critical role in regulating immunity in ADs [9] as well as cancer, infections, transplantation, and allergy [2]. The single-cell RNA sequencing transcriptional profiles of murine Bregs were first reported in 2021 [10]. Recent research has shown that Bregs driven by oxidative stress-initiated one-carbon metabolism alleviated pneumonia [11]. The protective effect of Bregs promoted by altered gut microbiota was identified in type 1 diabetes (T1D) in toll-like receptor (TLR) 9-deficient mice [12]. Additionally, Mauri *et al*. [13] have highlighted the therapeutic potential of thioredoxin by controlling Bregs.

## Phenotypes and effectors of Bregs

As illustrated in Supplementary Table 1, Bregs exhibiting various phenotypes assumes distinct roles under different conditions. Unlike FoxP3 for regulatory T cells, there is currently no specific transcriptional marker available to comprehensibly identify Bregs. Several Bregs subsets have been discovered to produce IL-10 (B10), yet there is no single phenotype that definitively identifies B10 cells [14]. B10 cells are typically enriched by the phenotype of CD19^hi^CD1d^hi^CD5^+^ in murine spleen and CD24^hi^CD27^+^ in the peripheral blood of humans [15]. In humans, although a Bregs subset displaying the CD19^+^CD1d^+^CD5^+^ phenotype has been observed, it is not classified as a B10 cell since this subset does not rely on IL-10 for its functionality [16].

Bregs exert immune suppression through the production of various effectors, including IL-10, TGF-β, and IL-35. IL-10 was identified as a marker of Bregs inhibitory function as early as the 2000s [7]. TGF-β has been shown to induce apoptosis of CD4^+^ T cells [17], promote anergy in CD8^+^ effector T cells [18], and facilitate the differentiation of CD4^+^ T cells to Treg cells [19]. IL-35, which is associated with Treg function [20], has been demonstrated to inhibit pathogenic T cells [21, 22] and macrophages [21] in independent studies. In addition to these three effectors, other inhibitory molecules expressed by Bregs were also detected, such as granzyme B (GrB), PD-L1, CD39 and CD73, aryl hydrocarbon receptor (AhR), etc [2].

## Impaired Bregs in ADs

Bregs are abundant and undergo maturation in the first 3 years of pediatric life [23]. Following this period, the frequency of CD19^+^CD24^hi^CD38^hi^ transitional Bregs (tBreg) gradually decreases with age, while there is an increase in the frequency of CD19^+^CD24^hi^CD27^+^ memory Bregs (mBreg) [23]. Approximately 25% of total B cells are comprised of CD19^+^CD24^hi^CD27^+^ B cells in healthy population, and nearly all B cell subsets have the potential to transition into Bregs phenotype in the context of inflammatory diseases [24]. Moreover, Lino *et al*. [25] identified a population of natural regulatory plasma cells (LAG-3^+^CD138^hi^) in the murine spleen and bone marrow, opposing to reactive plasmablasts.

**Figure 1.**
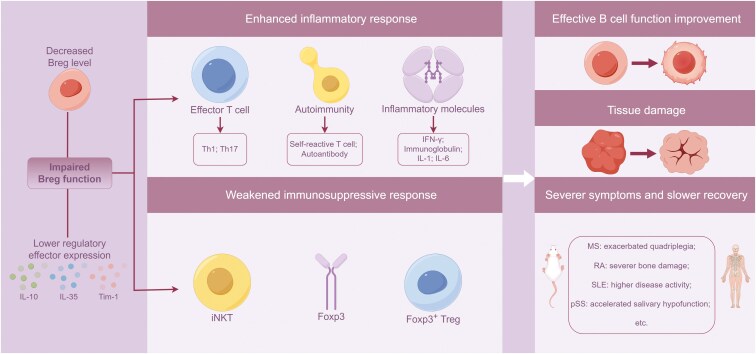
Impaired function and consequences of Bregs in health and disease. Impaired Bregs cell function is typically characterized by a reduction in Bregs cell quantity and/or diminished expression of regulatory effectors such as IL-10, IL-35, and Tim-1. This impairment leads to heightened autoimmunity and an increase in effector T cell subsets and inflammatory molecules. Additionally, the immunosuppressive capabilities of invariant natural killer T (iNKT) cells and FoxP3^+^ regulatory T cells (Tregs) are compromised, which is also accompanied by reduced FoxP3 expression. This cascade of events results in escalated effector B cell activity, tissue damage, exacerbation of autoimmune disease symptoms, and delayed recovery.

Given the central role of Bregs in the immune tolerance, their defects are long considered closely linked to the autoimmune pathogenesis (Fig. 1). It is believed that the impaired function of Bregs in the pathogenesis of systemic lupus erythematosus (SLE) can be attributed to disruptions in B cell activation, B cell co-stimulation, and cytokine signaling pathways [26]. The potentially key effector of Bregs, IL-10, has also been found to be negatively regulated by various transcription factors, such as the ETS family transcription factor (ETS-1) and T-box expressed in T cells (T-Bet) [27]. Other molecules, including major histocompatibility complex class Ⅱ transactivator (CⅡTA), poly (ADP) ribose polymerase 1 (PARP-1), B-cell lymphoma 3 (Bcl-3), IFN-γ, and signaling lymphocytic activation molecule family member 5 (SLAMF5), have also been shown to suppress IL-10 expression [27, 28]. Furthermore, a recent study has demonstrated that the deficiency of Hspa13, a heat shock protein (HSP) belonging to the HSP70 family, led to reduced IL-10 production, impaired marginal zone (MZ) B cells function, and exacerbation of SLE progression [29].

Impaired Bregs in patients with multiple sclerosis (MS) produced less IL-10 and failed to inhibit effector T cell function [30]. Bregs in patients with rheumatoid arthritis (RA) lost their ability to suppress Th17 development or induce the differentiation of T cells into suppressive Tregs [31]. Functionally defective Bregs isolated from SLE patients also produced less IL-10 and were refractory to CD40 stimulation [8]. This was associated with impaired in function and number of invariant natural killer T (iNKT) cells, which contributed to the maintenance of tolerance in autoimmunity, in SLE patients [32]. Consequently, self-reactive T cell activity and auto-antibody formation were found enhanced in patients with Bregs defects, resulting in severe auto-inflammatory sequelae [33]. Notably, patients experiencing flares of ADs have shown limited numerical recovery of Tregs and B10 cells following gene therapy [34]. However, in contrast, controversial results were reported in a separate study, including those with RA, SLE, Sjögren’s disease, MS, or autoimmune vesiculobullous skin disease, showing increased total frequencies of IL-10-competent B10 cells and progenitor B10 cells when compared with healthy controls (HC) [35].

Severer symptoms and delayed remission have been reported closely associated with deficiency of IL-10^+^ or IL-35^+^ Bregs in mice with experimental autoimmune encephalomyelitis, a mouse model of human MS [36]. Similarly, mice lacking of IL-10 or B10 cells displayed strong type 1 autoimmunity [7], increased Th1 and Th17, decreased FoxP3 expression and FoxP3^+^ Treg level [37]. Although negative regulators of Bregs response are not fully understood, the absence of Tim-1 was found responsible for reduced IL-10 production, leading to elevated IFN-γ, serum immunoglobulin, and autoantibodies [38]. Consistently, increased pro-inflammatory cytokines such as IL-1 and IL-6, augmented Th1 and Th17 responses, and decreased IL-10-producing FoxP3^+^ Treg cell counts were also reported in mice with Tim-1 defects [39]. Thus, we next summarized disease specific findings in Bregs in the context of autoimmunity (Table 1).

**Table 1.** Changes in Bregs and related molecules from human and mice with different AD

AD	Species	Breg			Regulatory molecules		Reference
		Phenotype	Level	Function	Effector	Level	
MS	Human	CD19^+^CD24^hi^CD38^hi^, CD19^+^CD24^low/neg^CD38^hi^	−		IL-10		[40]
		CD19^+^CD24^hi^CD38^hi^		−	IL-10	−	[30]
		CD19^+^CD24^hi^CD38^hi^	+		IL-10		[41]
		CD19^+^CD24^hi^CD27^+^CD38^hi^	−				
		CD19^+^CD24^hi^CD27^+^	=				
		CD19^+^CD20^+^CD27^+^Tim-1^+^TIGIT^+^		−			[42]
	Mouse	CD1d^hi^CD5^+^	+		IL-10		[44]
		CD138^+^CD44^hi^	+		IL-10		[36]
		CD5^+^CD11b^+^CXCR4^+^LAG3^+^PD-1^+^PD-L1^+^	+		IL-27	+	[46]
RA	Human	CD19^+^CD27^+^IL-10^+^	−	−	IL-10		[47]
		CD19^+^CD24^hi^CD38^hi^	−	−	IL-10		[31]
		CD19^+^PD-L1^+^, CD24^hi^CD38^-^PD-L1^+^, CD24^hi^CD38^hi^PD-L1^+^	−		PD-L1		[48]
		CD19^+^GrB^+^	−	−	GrB		[50]
		CD19^+^LAG-3^+^	−	−	LAG-3		[49]
	Mouse	CD5^+^FasL^+^	−				[55]
		CD1d^hi^CD5^+^	-→+		IL-10		[54]
		CD19^+^CD21^hi^CD23^+^IgM^hi^IL-10^+^	+		IL-10		[57]
SLE	Human	CD19^+^CD24^hi^CD38^hi^		−	IL-10	−	[8]
		CD19^+^CD5^+^CD1d^hi^	+		IL-10	+	[62]
		CD19^+^CD24^hi^CD38^hi^	−				[58]
		CD19^+^CD5^+^	−				[61]
		CD19^+^IL-10^+^			IL-10	+	
		CD19^+^IL-35^+^			IL-35	−	
		CD24^hi^CD38^hi^, CD24^hi^CD27^+^	−				[64]
		CD24^dim/-^CD27^lo/-^CD38^lo/-^CD69^+/hi^			IL-10		
		CD19^+^CD24^hi^CD27^+^	−				[101]
					IL-10	+	
					IL-35	−	
		CD19^+^GrB^+^	−	−	GrB		[59]
	Mouse	CD19^+^CD5^+^CD1d^hi^	+		IL-10		[102]
		CD19^+^CD21^hi^CD23^+^IgM^hi^IL-10^+^	−	−	IL-10		[67]
		CD21^hi^CD23^low/neg^CD1d^hi^	−				[103]
		CD21^hi^CD23^+^IgM^hi^	−				
		CD5^+^CD1d^−^CD38^+^GL7^-^IL-10^+^	+		IL-10		[68]
		CD19^+^GrB^+^	−	−	GrB		[65]
		CD19^+^CD5^+^CD1d^+^	=		IL-10	+	[66]
					IL-35	−	
					TGF-β	−	
SjD	Human	CD19^+^CD24^+^CD38^hi^	−	−	IL-10	−	[71]
		CD19^+^CD5^+^GrB^+^	+	+	IL-21R, GrB	+	[73]
		CD19^+^CD24^hi^CD27^+^	−		IL-10		[9]
		CD19^+^EBI3^+^	+				[72]
					IL-35, EBI3, P35	−	
	Mouse	CD19^+^CD1d^hi^CD5^+^	−	−	IL-10	−	[71]
T1D	Human	CD19^+^CD24^hi^CD38^hi^, CD19^+^CD24^hi^CD38^hi^Tim^+^, CD19^+^CD24^hi^CD38^hi^IL-10^+^	−	−	Tim, IL-10	−	[104]
		CD19^+^CD24^+^CD40^+^CD38^+^	−				[75]
		CD19^+^CD24^+^CD40^+^CD38^+^IL-35^+^	−		IL-35		
		CD19^+^CD24^+^CD40^+^CD38^+^IL-10^+^	=		IL-10		
		CD19^+^CD25^hi^	−				[76]
	Mouse	CD19^+^CD1d^hi^CD5^+^	−	−	IL-10	−	[74]
		CD19^+^CD1d^+^CD5^+^	−/+→-				[75]
		CD19^+^CD1d^+^CD5^+^IL-35^+^	+→-		IL-35		
		CD19^+^CD138^+^IL-35^+^	−		IL-35		
		CD19^+^CD1d^+^CD5^+^IL-10^+^	=/+		IL-10		
LADA	Human	CD19^+^CD27^hi^CD38^hi^	+				[77]
		CD19^+^CD24^hi^CD27^+^	+		IL-10		
MG	Human	CD19^+^CD24^hi^CD38^hi^	−	−	IL-10		[85]
		CD19^+^IL-10^+^	−				
		CD19^+^CD38^-^IL-10^+^FoxP3^+^	+		IL-10, FoxP3		[86]
		CD19^+^CD38^+^IL-10^+^FoxP3^+^	−				
SSc	Human	CD19^+^IL-10^+^	−				[79]
		CD19^+^CD24^hi^CD38^hi^	−				[78]
		CD19^+^CD24^hi^CD27^+^					
Ps	Human	CD19^+^CD24^hi^CD38^hi^	=		IL-10	+	[89]
IMN	Human	CD19^+^CD24^hi^CD38^hi^	+	−			[84]
NMOSD	Human	CD19^+^CD24^hi^CD38^hi^	−	−	IL-10	−	[82]
ITP	Human	CD19^+^CD24^hi^CD38^hi^	−	−			[80]
		CD19^+^IL-10^+^	−	−	IL-10	−	
		CD19^+^TGF-β^+^	−	−	TGF-β	−	
AA	Human	CD19^+^CD24^hi^CD38^hi^	−				[81]
TAO	Human	CD19^+^IL-35^+^	=				[87]
		CD19^+^CD24^hi^CD38^hi^	−		IL-10		[88]

Abbreviations: IL, Interleukin; CXCR, C-X-C chemokine receptor; LAG-3, Lymphocyte Activation Gene-3; PD-1, Programmed cell death 1; PD-L1, Programmed cell death ligand 1; GrB, Granzyme B; FasL, Fas Ligand; Ig, Immunoglobulin; TGF-β, Transforming growth factor-Beta; SjD, Sjögren syndrome; EBI3, Epstein-Barr virus-induced gene 3; T1D, Type 1 diabetes; Tim-1, T cell Ig and mucin domain-1; FoxP3, Forkhead box P3; Ps, Psoriasis.

### Multiple sclerosis

Converging evidences have demonstrated deficiencies in CD19^+^CD24^hi^CD38^hi^ tBregs [40] and IL-10 production [30] in patients with MS compared with the HC. Impaired function of CD19^+^CD24^hi^CD38^hi^ Bregs was reflected by reduced IL-10 production and their inability to suppress effector T-cell function [30]. A prospective study of tBregs subsets has revealed an increased frequency of CD19^+^CD24^hi^CD38^hi^ phenotype but a decreased proportion of differentiated CD27^+^ cells within this subset in patients with MS, suggesting a potential shift between Bregs cell maturation and effector memory B cells during the progression of MS [41]. Additionally, a population of CD19^+^CD20^+^CD27^+^ memory Bregs, co-expressing Tim-1 and TIGIT, was identified functionally impaired in MS [42].

Supporting evidence from the murine EAE model has demonstrated that the depletion of B10 cells can exacerbate the symptoms such as abnormal gait and quadriplegia [36] with hindered disease remission [7]. Bregs have been shown to limit autoimmune inflammation [36], polarize M2 macrophages, promote oligodendrocytes, and enhance remyelination [43]. The transfer of Bregs has been demonstrated to reduce the initiation of EAE [44] and reversed clinical exacerbation [45]. The absence of IL-35 has also been found to impede disease recovery from T cell-mediated EAE [21]. Furthermore, pathogenic Th17 and Th1 cells were inhibited by IL-35 secreted by Bregs in mouse model of experimental autoimmune uveoretinitis (EAU) [22]. Of note, the neuroinflammation in both EAE and EAU can be suppressed by IL-27-producing B-1a cell subset [46].

### Rheumatoid arthritis

Bregs from patients with RA expressed less IL-10^+^ [47], PD-L1^+^ [48], LAG3^+^ [49] and GrB, as well as lower levels of IL-21R [50] when compared with HC. The LAG3^+^ and GrB^+^ Bregs were negatively associated with the aggravated RA symptoms [49]. In addition, B10 cells from RA patients were found to enhance the differentiation of naive T cells into Th1 cells compared with the counterparts from HC. In this process, PD-L2, a PD-1 ligand that inhibits PD-L1 and promotes Th1 differentiation, was found to be overexpressed on the RA B10 cells [51]. In this regard, recent advances have shown a critical role of AhR signaling in Bregs differentiation, which may potentiate the application of pharmaceutical agonists to restrain disease activities in RA [52].

The importance of Bregs in RA immunosuppression has been elucidated in murine models. Adoptive transfer of B10 cells effectively prevented murine RA [53] and alleviated damage in bone and cartilage, associated with a decrease in pathogenic Th17 cells [54]. Similar to the findings in MS, B-1a cells from the spleen were found to induce apoptosis in CD4^+^ T cells and suppress RA in mice [55]. MZ B cells, influenced by apoptotic cells, can enhance the secretion of IL-10, thereby preventing severe inflammatory RA and reducing pathogenic autoantibodies [56]. Type 2 MZ precursor (T2-MZP) B cells are a minor subset during B cell development. Recent studies have reported their immunosuppressive properties [57], which reversed the decrease in FoxP3^+^ Tregs, and suppressed inflammatory Th1 and Th17 cells in B10-deficient mice [37].

### Systemic lupus erythematosus

Similarly, emerging evidence has demonstrated the impaired suppressive function of Bregs from patients with SLE [58], in particular in those patients with higher disease activities and erythrocyte sedimentation rates [59]. Mauri *et al.* reported that plasmacytoid dendritic cells (pDCs) could promote Bregs in HC, but not in the context of SLE [60]. In contrast, decreased level of IL-35 and IL-35^+^ Bregs has been observed, while higher levels of IL-10 were detected in SLE patients with a lower percentage of IL-10^+^ Bregs, indicating the protective role of IL-35 and IL-35^+^ Bregs in SLE [61]. Furthermore, the percentage of Bregs and the production of IL-10 were elevated in SLE patients [62]. T follicular helper (Tfh) cells are a major source of IL-21, which can enhance IL-10 production and Bregs cell differentiation. Thus, the expansion of Tfh has been positively correlated with the increased Bregs phenotype in turn [62]. However, in contrast, IL-10 also facilitated autoantibody production and extrafollicular autoimmune responses in patients with active SLE [63]. IL-10^+^ Bregs in SLE patients were deemed to be aggressively inflammatory, eliciting dual roles in inducing pathogenic CD4^+^ T cell response and the shift away from CD8^+^ T cell tolerance [64].

Compiling data from murine lupus models have demonstrated the distorted Bregs response during disease progression [65]. In addition to reduced cell numbers, increased IL-10 but decreased IL-35 and TGF-β in Bregs were detected in SLE mice [66]. Study has shown that T2-MZP Bregs inhibited Th1 responses by promoting the differentiation of IL-10^+^CD4^+^ T cells and conveying a regulatory effect to CD4^+^ T cells [67]. B10 cells can induce the expansion of functional Treg cells, as adoptive transfer of induced Treg cells can effectively inhibit lupus progression and prolong survival of lupus mice [68]. Nevertheless, different from the above models, studies have also reported increased production of IL-10 by B cells in lupus mice [69]. The hyperactive IL-10 response was attributed to MZ B cells from lupus mic, which were also the major responder cells to CpG-oligodeoxynucleotides stimulation [69]. Interestingly, although increased CD38 expression has been identified as one of the biomarkers of Bregs in both humans and mice [8], the frequency of B10 cells (CD19^+^CD1d^hi^CD5^+^) was found to be significantly higher in CD38 deficient lupus mice, when compared with those wild-type counterparts [70]. This may be associated with the alteration of peritoneal pDCs and IFN-α in CD38 deficient mice [70].

### Sjögren’s disease

The declines in Bregs were also observed in both Sjögren’s disease (SjD, or Sjögren’s syndrome) patients and mice with experimental Sjögren’s syndrome (ESS) mice, accompanied by higher autoantibodies and exacerbated salivary pathology [71]. B cell-derived IL-10 played a critical role in restraining the differentiation and generation of Tfh cells during SjD development [71]. Different from the findings in SLE, a negative correlation between Tfh cells and Bregs has been clarified by lower IL-10-producing capacity in Bregs [71]. In addition, plasma IL-35 levels in SjD patients were also significantly lower than those in HC [72]. Besides, a potential protective regulatory effect of IL-21-induced GrB^+^CD19^+^CD5^+^ cells in human peripheral blood was suggested, which was increased in patients with SjD [73].

### Autoimmune diabetes

The frequencies of total Bregs in the peripheral blood mononuclear cells (PBMCs), including Tim-1^+^ Bregs, IL-35^+^ Bregs, and IL-10^+^ Bregs were found decreased in patients with type 1 diabetes (T1D) compared with those in the HC [74, 75]. The defects of IL-10^+^ Breg [74] and IL-35^+^ Breg [75] were considered as the results from T1D progression. A subset of CD25^hi^ Bregs was reported positively correlated with Treg cells in HC but significantly altered in patients with T1D [76]. However, plasmablasts and B10 cells demonstrated an increasing trend in Latent autoimmune diabetes in adults (LADA) [77].

### Other autoimmune disorders

Likewise, the autoimmune conditions were frequently associated with the defects in the numbers and functions in Bregs reported from many studies. For instance, the reduction of CD24^hi^CD27^+^ subset [78] in patients with systemic sclerosis (SSc) may result in decreased circulating Bregs when compared with those in HC [79]; Weakened immunosuppressive capacity of Bregs in the bone marrows was detected in patients with primary immune thrombocytopenia (ITP), while functional Bregs therapy can markedly alleviate thrombocytopenia in mice [80]. Similar results have also been detected in patients with alopecia areata (AA) [81], neuromyelitis optica spectrum disorder (NMOSD) [82], and Behcet disease (BD) [83]. In patients with idiopathic membranous nephropathy (IMN), the frequency of CD19^+^CD24^hi^CD38^hi^ Bregs was increased, but their regulatory function was significantly impaired [84].

Several studies on ADs have yielded divergent conclusions regarding alterations in the quantity and function of Bregs. Specifically, reports have documented a reduction in Bregs numbers and functional disturbances in patients with myasthenia gravis (MG) [85]. Another study reported elevated levels of a rare subset of CD38^-^FoxP3^+^IL-10^+^ Bregs and decreased levels of CD38^+^FoxP3^+^IL-10^+^ Bregs in pediatric patients with Graves’ disease [86]. In patients with thyroid-associated ophthalmopathy (TAO), one study observed a higher percentage of circulating IL-35^+^ Bregs following stimulation with CpG compared with HC [87], whereas their suppressive capacity was defective during the disease active phase [88]. In contrast, enhanced IL-10 production was detected in Bregs from patients with psoriasis [89]. Their regulatory function was intact as in mouse models of experimental psoriasis, which inhibit disease pathology in mice with expanded Treg cells and diminished Th17 cell differentiation [90].

### Potentially clinical significance of Bregs in autoimmune treatment

In a longitudinal study, it was observed that the deficiency of CD19^+^CD24^hi^CD38^hi^ Bregs was evident in MS patients during disease relapse but was restored during remission [91]. Other studies have reported increased Bregs numbers and IL-10 levels in response to targeted treatments for patients experiencing recurrent MS [40, 92]. Similarly, a significant reduction in PD-L1^+^ Bregs was observed in RA patients, which was restored following successful treatment [48]. This suggested that variations in the levels of Bregs may be closely associated with the patient status, and thus can serve as a promising biomarker for monitoring disease progression and treatment response, in terms of relapse and remission. Further confirmation would require additional clinical trials with a multi-center approach and larger sample size to validate Bregs as a potential biomarker.

In conclusion, the role of Bregs in ADs appears to be complex and variable. While many studies report alterations in Bregs quantity and function, these changes are not consistent across different diseases or even within the same disease. Clinical evidence highlights the heterogeneous nature of Bregs involvement in autoimmune pathogenesis and underscore the need for further research to elucidate the mechanisms driving these divergent trends.

## Bregs as a therapeutic target in the treatment of ADs

A recent comprehensive review has summarized a series of potential therapies for ADs treatment by targeting Bregs [93]. These therapies aim to modulate Bregs function and numbers to restore immune balance and alleviate disease symptoms. Additionally, Bao *et al*. further developed a protocol using a PKA-CREB agonist to induce human CD1c^+^ Bregs *in vitro*. These CD1c^+^ Bregs effectively suppress the proliferation of PBMCs and reduce the secretion of inflammatory cytokines by T cells. When transferred into a humanized mouse model of graft versus host disease, the induced human CD1c^+^ Bregs strongly alleviated the symptoms in mice [94]. Moreover, another approach to promote human Bregs is recently introduced by silencing miR-29a-3p, a regulatory microRNA involved in the differentiation of mBregs. This was supported by the inhibition of miR-29a-3p which resulted in a significant enhancement of the differentiation and immunosuppressive function of CD19^+^CD24^hi^CD27^+^ mBregs in liver transplant recipients [95].

Recent efforts are also advancing the *in vivo* restoration of murine Bregs under specific treatments. For instance, the sphingosine 1-phosphate antagonist fingolimod has been shown to effectively reduce germinal center B cells and increase Bregs, leading to improvements in murine SjD [96]. Similarly, acteoside from a medicinal herb *Radix Rehmanniae*, can effectively enhance the function of both human and murine B10 cells, reduce Th17 and Tfh and then alleviate the pathology in ESS mice [97]. Additionally, esculentoside A, a compound derived from the traditional Chinese medicine *Phytolacca esculenta*, significantly increases CD19^+^IL-35^+^ Bregs and IL-35 levels while decreasing IL-17 levels, thereby inhibiting disease progression in mice with lupus nephritis [98]. Treatment with recombinant IL-35 has been found to enhance IL-35^+^ Bregs, IL-35^+^IL-10^+^ Bregs, and LAG-3^+^ Tregs in the lungs of asthmatic mice [99]. Furthermore, CpG treatment has been able to restore IL-10 expression in the intestines of food-allergic mice, promoting immunotherapy for food allergy [100].

Taken together, improving the amount and regulatory function of Bregs, or *ex vivo* expansion of Bregs followed by cell therapy, are currently considered as viable strategies when targeting Bregs in the drug development (Table 2). Furthermore, regulating the Bregs-featured effector molecules, or related pathways, may also be promising strategies.

**Table 2.** Therapeutic strategies targeting Bregs

Strategies	Description	References
Improving Bregs function	Butyrate supplementation: activating AhR and enhancing Breg function while inhibiting the differentiation of germinal center (GC) B cells and plasma cells, and suppressing RA	[52]
	*Radix Rehmanniae*: enhancing the function of both human and murine B10 cells, reducing Th17 and Tfh.	[97]
	CpG: restoring IL-10 expression	[100]
Improving Bregs expansion	Thioredoxin stimulation: restoring Bregs from patients with SLE to healthy levels	[13]
	Blocking SLAMF5 (CD84): mitigating EAE and increasing Breg levels	[28]
	Thymosin-α1: reducing IL-6, IL-8, and IL-1β while increasing IL-10 and IL-35; enhancing CD19^+^CD24^+^CD38^hi^ and CD24^low/neg^CD38^hi^ Bregs	[40]
	Recombinant mouse IL-35: preventing hyperglycemia, increasing Breg cells and IL-35 Breg cells, decreasing the proportions of IFN-γ^+^ cells among Bregs	[75]
	Alemtuzumab: shifting the distribution of B cells towards naïve phenotype and restoring CD19^+^CD24^hi^CD38^hi^ and CD19^+^PD-L1^hi^ Breg deficiency	[91]
	Fingolimod: antagonizing sphingosine 1-phosphate to reduce Th17 and germinal center B cells, increase Bregs and Tregs	[96]
	Recombinant IL-35: enhancing IL-35^+^ Bregs, IL-35^+^IL-10^+^ Bregs, and LAG-3^+^ Tregs	[99]
Improving both function and expansion of Bregs	Transferring altered gut microbiota: promoting Breg cell differentiation and IL-10 secretion	[12]
	B cell depletion therapy (BCDT): increasing Breg amount; post-BCDT repopulation of CD24^hi^CD38^hi^ B cells: restoring IL-10 production, suppressing IFN-γ and IL-17 production by CD4 T cells	[82]
	Fingolimod: reducing CXCR4-mediated B cell migration, increasing TGF-β^+^ Bregs and inducing Breg-mediated anti-inflammatory immune repertoire	[92]
	Silencing miR-29a-3p microRNA: enhancing the differentiation and immunosuppressive function of CD19^+^CD24^hi^CD27^+^ mBregs	[95]
	Esculentoside A from *Phytolacca esculenta:* increasing CD19^+^IL-35^+^ Bregs and IL-35 levels, decreasing IL-17 level	[98]
Ex vivo expansion or activation of Bregs followed by cell therapy	LPS: improving Fas ligand expression and TGF-beta secretion of B cells, transfer of which inhibited spontaneous Th1 autoimmunity and prevented autoimmune diabetes	[17]
	IL-35: inducing Bregs and promoting their conversion to IL-35^+^ and IL-10^+^ Breg, transfer of which suppressed EAU, inhibited Th17 and Th1 cells while promoting Tregs	[22]
	Agonistic anti-CD40: enriching Bregs *in vitro*, transfer of which inhibited Th1 responses by promoting the differentiation of IL-10^+^CD4^+^ T cells and conveying a regulatory effect to CD4^+^ T cells	[67]
	PKA-CREB agonist: inducing human CD1c^+^ Bregs with the ability to suppress PBMCs and inflammatory T cells, transfer of which alleviated graft versus host disease	[94]

## Conclusion

In this review, we have summarized the alterations in Breg phenotypes across various ADs. Given their dynamic changes during different treatment stages in patients with autoimmune disorders, Bregs may hold significant clinical relevance, potentially serving as promising biomarkers for disease diagnosis and prognosis.

Furthermore, therapeutic strategies targeting Bregs have been explored, showing improved clinical outcomes in ADs. First, the underlying mechanism of Breg cell functionality paves the way to develop medication that achieves immune tolerance in a more precise way. Furthermore, industrial grade of *ex vivo* Breg expansion may shed new light in the drug development of cell therapy. Meanwhile, although it is not yet formally discussed, in spite of efficacy, whether there will be potential safety issue of Breg-targeted treatments shall be also taken into account in the future studies.

## Supplementary Material

ltaf020_suppl_Supplementary_Table_S1

## Data Availability

Data will be made available on request.

## Permission to reproduce

All the figures in the manuscript were drawn by Figdraw.
