# Supplementary material for: Heterogeneity of regulatory B cells in autoimmune diseases: implications for immune equilibrium and therapeutic strategies
Source: Immunother Adv. 2025 May 8;5(1):ltaf020. doi: 10.1093/immadv/ltaf020 (PMC12128196; doi:10.1093/immadv/ltaf020)
Supplement: ltaf020_suppl_Supplementary_Table_S1 [file ltaf020_suppl_supplementary_table_s1.docx]

Supplementary Table 1. Bregs phenotypes and functions in different diseases or models

| Type of Breg | Phenotypes | Effectors | Functions/Findings | Conditions | | References |
| --- | --- | --- | --- | --- | --- | --- |
|  |  |  |  | Species | Diseases/Models |  |
| Immature cells | CD19^+^CD24^+^CD38^hi^ | IL-10 | Suppress autologous Tfh cell expansion | Human | SjD | ^[1]^ |
|  | CD19^+^CD24^hi^CD38^hi^ | IL-10 | Suppress the differentiation of Th1 cells | Human | SLE | ^[2]^ |
|  | CD19^+^CD24^hi^CD38^hi^ | IL-10 | Suppress CD4^+^CD25^-^ T cell proliferation and the release of IFN-γ and TNF-α by these cells; inhibited naive T cell differentiation into Th1 and Th17 cells and converted CD4^+^CD25^-^ T cells into Tregs | Human | RA | ^[3]^ |
| BR1 cells | CD19^+^CD25^hi^CD71^hi^CD73^lo^ | IL-10 | Suppress antigen-specific CD4^+^ T-cell proliferation; up-regulate IgG4 production | Human | Allergy | ^[4]^ |
|  | CD19^+^CD73^−^CD25^+^CD71^+^IL-10^+^ | IL-10 | Expand after high-dose allergen exposure | Human | Allergy | ^[5]^ |
| tBreg | CD19^+^CD24^hi^CD38^hi^CD27^+^ | IL-10 | Inhibit TNF-α and IFN-γ production by T cells | Human | ADs, HIV, rejection and healthy donors | ^[6]^ |
|  | CD19^+^CD24^hi^CD38^hi^IgM^lo^IgD^lo^ | IL-10 | Reduce CD4^+^ T-cell proliferation | Human |  |  |
| mBreg | CD19^+^CD20^+^CD27^+^Tim-1^+^ TIGIT^+^ | IL-10 | Suppress T cell proliferation and IL-17A, IFNγ and TNFα production | Human | MS | ^[7]^ |
| GrB^+^ B cells | CD19^+^CD38^+^CD1d^+^IgM^+^CD147^+^ | GrB, IL-10, CD25, IDO | Suppress CD4^+^ T-cell proliferation | Human | Five various solid epithelial cancers | ^[8]^ |
|  | CD38^+^CD19^+^ | IL-10, IL-35, TGF-β, GrB, Tim-1, CD5, CD21 | Be associated with increased FoxP3^+^CD4^+^ Tregs and decreased activated NK cells | Human | Pancreatic ductal adenocarcinoma | ^[9]^ |
|  | CD5^+^CD38^+^CD27^+^CD138^+^CD19^+^ | GrB | Directly inhibit the proliferation, migration, and invasion of hepatocellular carcinoma cells | Human | Liver transplant recipients with hepatocellular carcinoma recurrence | ^[10]^ |
| T2-MZP | CD19^+^CD21^hi^CD23^hi^CD24^hi^ | IL-10 | Inhibit antigen-specific T cell activation; reduce cells exhibiting Th1-type functional responses | Mice | Experimental arthritis | ^[11]^ |
|  | CD19^+^CD21^hi^CD23^hi^CD24^hi^ | IL-10 | Induce FoxP3^+^CD4^+^ T cells and suppress TNF-α^+^CD4^+^ T cells | Mice | Experimental arthritis | ^[12]^ |
|  | CD19^+^CD1d^hi^CD5^+^CD21^hi^CD23^+^IL-10^+^IgD^+^IgM^hi^ | IL-10 | Induce pulmonary infiltration of CD4^+^CD25^+^ FoxP3^+^ Tregs | Mice | Allergic airway inflammation | ^[13]^ |
| B10 cells | CD19^+^CD24^hi^CD27^+^CD148^hi^CD48^hi^ | IL-10 | Negatively regulate monocyte cytokine production | Human | ADs | ^[14]^ |
|  | CD24^int^CD27^−^CD38^−^CD69^+/hi^ | IL-10 | Display aggressive inflammatory features, which shift the functions away from inducing CD8^+^ T cell tolerance and cause them to induce a pathogenic CD4^+^ T cell response | Human | SLE | ^[15]^ |
|  | CD19^+^CD24^hi^CD38^hi^CD39^-^ | IL-10 | Limit T helper-cell proliferation, type-1 cytokine production, and T effector-cell survival; augment CD4^+^FOXP3^+^ Treg generation | Human | Breast cancer | ^[16]^ |
|  | CD19^+^CD5^+^CD1d^hi^ | IL-10 | Inhibit T cell proliferation and T cells-producing IL-17 | Mice | MS (EAE) | ^[17]^ |
|  | CD19^+^CD5^+^CD1d^hi^ | IL-10 | Inhibit intestinal injury | Mice | Experimental ulcerative colitis | ^[18]^ |
|  | CD19^+^CD5^+^CD1d^hi^ | IL-10 | Suppress Th17 cell generation | Mice | Experimental arthritis | ^[19]^ |
|  | CD19^+^CD5^+^CD1d^hi^IL-10^+^ | IL-10 | Suppress Tfh cell differentiation and restrain the effector Tfh cell response | Mice | ESS | ^[1]^ |
| MZ B cells | CD19+CD21^hi^CD23^−^ | IL-10 | Negatively regulate IL-12p40 and inhibit NK  cells-producing IFN-γ | Mice | SLE | ^[20]^ |
|  | CD19+CD21^hi^CD23^−^ | IL-10 | Induce an IL-10-secreting T Cell population | Mice | Experimental arthritis | ^[21]^ |
| Tim-1 B cells | Tim-1^+^CD19^+^ | IL-10 | Promote a Th2 response; enhance IL-10 expression on Tregs; prolong allograft survival | Mice | Islet transplantation | ^[22]^ |
| Plasma cells | CD19^+^CD20^lo^B220^lo^IgA^+^ | IgA, IL-10, PD-L1 | Induce CD8^+^ cell exhaustion and suppress anti-tumor CTL responses; impede T-cell-dependent immunogenic chemotherapy | Human | Prostate cancer | ^[23]^ |
|  | CD19^+^CD138^+^ | IL-10 | Mediate part of the suppressive functions resulting from MyD88 signaling in B cells | Mice | Salmonella typhimurium infection | ^[24]^ |
|  | IgM^+^CD138^hi^TACI^+^CXCR4^+^CD1d^int^Tim1^int^ | IL-10, IL-35 | Inhibit anti-microbial immunity | Mice | Salmonella infection | ^[25]^ |
|  | CD19^+^CD138^+^ | IL-10, IL-35 | Reduce the accumulation of pathogenic cells in the target organ; regulate T cell-mediated autoimmunity; regulate the function of B cells as antigen-presenting cells | Mice | MS (EAE) |  |
|  | IL-10^+^LAG-3^+^CD138^hi^ | LAG-3, CD200, PD-L1 , PD-L2 | Rapidly provide a first layer of B cell-mediated immune regulation in response to toll-like receptor signals; inhibit host defense | Mice | Salmonella typhimurium infection | ^[26]^ |
| Plasmablasts | CD27^int^CD38^+^ | IL-10 | Produce IL-10; secrete Ig | Human | HD | ^[27]^ |
|  | CD138^+^CD44^hi^ | IL-10 | Inhibit dendritic cell function to generate pathogenic T cells | Mice | MS (EAE) |  |
| B1a cells | CD19^+^CD5^+^CD1d^+^ | Not dependent on IL-10 | Inhibit Mycobacterium tuberculosis antigen-specific IL-22 production | Human | Tuberculosis | ^[28]^ |
|  | CD19^+^CD5^+^CD1d^+^ |  | Inhibit Th17, but not Th1, cell activation |  |  | ^[29]^ |
|  | CD5^+^CD11b^+^CXCR4^+^LAG3^+^PD-1^+^PD-L1^+^ | IL-10, IL-35, IL-27, LAG-3, PD-1 | Up-regulate inhibitory receptors (LAG-3, PD-1); suppressTh17/Th1 responses; propagate inhibitory signals that convert conventional B cells to regulatory lymphocytes that secrete IL-10 and/or IL-35 | Human/Mice | HD/EAE, EAU | ^[30]^ |
|  | CD5^+^CD19^+^ | IL-10 | Negatively regulated type I IFNs and the inflammatory responses of pDCs and cDCs | Human/Mice | Neonatal inflammation | ^[31]^ |
| CD9^+^ cells | CD19^+^CD9^+^ | IL-10 | Induce effector T cell cycle arrest in sub G_0_/G_1_, leading to apoptosis in an IL-10-dependent manner | Human/Mice | Asthma | ^[32]^ |
|  | CD19^+^CD22^+^CD9^+^ | IL-10 | Suppress CD4^+^ T cell activities in an antigen-specific manner; CD22 mediates the effects of Galectin-9 to promote immunotherapy for allergic diseases by inducing B10 cells | Human/Mice | Allergic rhinitis | ^[33]^ |
| LARS B cells | CD19^+^IgD^−^ CD38^+^CD27^+^ | TGF-β1 | Promote CRC progression: the enrichment of LARS B cells in the tumor tissue was associated with the progression and prognosis of colorectal tumors | Human | CRC | ^[34]^ |
|  | CD19^+^B220^−^CD20^−^IgD^−^CD138^−^PRDM1^+^PAX5^+/lo^FAS^−^GL7^−^ | TGF-β1 | Drive the differentiation of CD4^+^ T cells into Treg cells, leading to immunosuppressive tumor environment and tumor evasion | Mice | CRC |  |
| CD25^+^ cells | CD19^+^CD25^+^ | TGF-β1 | Be positively associated with renal function, TGF-ß1 and absolute Treg counts | Human | Renal transplant recipients | ^[35]^ |
|  | CD19^+^CD25^hi^CD27^hi^CD86^hi^CD1d^hi^IL-10^hi^TGF-β^hi^ | IL-10, TGF-β | Suppress CD4^+^ T cells proliferation and enhance FoxP3 and CTLA-4 expression in Tregs | Human | HD | ^[36]^ |
|  | CD19^+^CD25^+^ | IL-10, IL-35, TGF-β | Reduce alveolar bone destruction and local Tregs, proportion, especially the local level of IFN-γ and IL-17; remedy the pathological change in the proportion of IL-1β and Th1/Th17 in local lesions | Mice | Experimental periodontitis | ^[37]^ |
|  | CD19^+^CD81^+^CD27^+^CD25^+^PD-L1^hi^ | IL-10;TGF-β | Inhibit T cell responses | Mice | Fibrosarcoma | ^[38]^ |
| Unclassified Breg | CD19^+^CD24^hi^CD27^+^ | IL-6, TNF-α | Enhance the acquisition of multidrug resistance and stem-like features of breast cancer cells | Human | Breast cancer | ^[39]^ |
|  | CD5^+^CD19^+^FasL^+^ | FasL | B cells could preferentially regulate the production of IL-17 versus IFNγ in a FasL-dependent manner | Mice | Experimental arthritis | ^[40]^ |
|  | CD5^+^CD19^+^CX3CR1^+^ | TGF-β | Induce Tregs in the intestine; suppress food allergy-related Th2 pattern inflammation | Mice | Food allergy-induced intestinal inflammation | ^[41]^ |

Abbreviation: Bregs, Regulatory B cells; IL, Interleukin; Tfh, T Follicular Helper cell; SjD, Sjögren syndrome; Th, T helper; SLE, Systemic lupus erythematosus; IFN-γ, Interferon-gamma; TNF-α, Tumor Necrosis Factor-alpha; Tregs, Regulatory T cells; RA, Rheumatoid arthritis; BR1 cells, B regulatory 1 cells; Ig, Immunoglobulin; tBreg, Transitional Bregs cells; tBreg, Transitional Bregs cells; ADs, Autoimmune diseases; HIV, Human Immunodeficiency Virus; mBreg, Memory Bregs cells; Tim-1, T cell Ig and mucin domain-1; TIGIT, T cell immune receptor with Ig and ITIM domains; MS, Multiple sclerosis; GrB, Granzyme B; IDO, Indoleamine-2,3-dioxygenase; TGF-β, Transforming growth factor-Beta; FoxP3, Forkhead box P3; NK cells, Natural killer cell; T2-MZP, Transitional 2 marginal-zone precursor; B10 cells, IL-10-producing Bregs cells; EAE, Experimental autoimmune encephalomyelitis; ESS, Experimental Sjögren's syndrome; MZ B cells, Marginal-zone B cells; PD-L1, Programmed cell death ligand 1; CTL, Cytotoxic T Lymphocyte; MyD88, Myeloid differentiation primary response gene 88; TACI, Transmembrane activator and calcium modulator and cyclophilin ligand interactor; CXCR, C-X-C chemokine receptor; LAG-3, Lymphocyte Activation Gene-3; PD-L2, Programmed cell death ligand 2; PD-1, Programmed Death Receptor 1; HD, Healthy donors; EAU, Experimental autoimmune uveitis; pDCs, Plasmacytoid dendritic cells; cDCs, Conventional dendritic cells; LARS B, Leucine-tRNA-synthase-2-expressing B cell; CRC, Colorectal cancer; PRDM1, PR-domain containing 1; PAX5, Paired box protein-5; CTLA-4, Cytotoxic T Lymphocyte-Associated Protein 4; FasL, Fas Ligand.

Supplementary Reference

[1] Lin X, Wang X, Xiao F, et al. IL-10-producing regulatory B cells restrain the T follicular helper cell response in primary Sjogren's syndrome [J]. Cell Mol Immunol, 2019,16(12): 921-931. doi: 10.1038/s41423-019-0227-z

[2] Blair PA, Norena LY, Flores-Borja F, et al. CD19(+)CD24(hi)CD38(hi) B cells exhibit regulatory capacity in healthy individuals but are functionally impaired in systemic Lupus Erythematosus patients [J]. Immunity, 2010,32(1): 129-140. doi: 10.1016/j.immuni.2009.11.009

[3] Flores-Borja F, Bosma A, Ng D, et al. CD19+CD24hiCD38hi B cells maintain regulatory T cells while limiting TH1 and TH17 differentiation [J]. Science Translational Medicine, 2013,5(173). doi: 10.1126/scitranslmed.3005407

[4] van de Veen W, Stanic B, Yaman G, et al. IgG4 production is confined to human IL-10-producing regulatory B cells that suppress antigen-specific immune responses [J]. Journal of Allergy and Clinical Immunology, 2013,131(4): 1204-1212. doi: 10.1016/j.jaci.2013.01.014

[5] Boonpiyathad T, Meyer N, Moniuszko M, et al. High-dose bee venom exposure induces similar tolerogenic B-cell responses in allergic patients and healthy beekeepers [J]. Allergy, 2017,72(3): 407-415. doi: 10.1111/all.12966

[6] Simon Q, Pers JO, Cornec D, et al. In-depth characterization of CD24(high)CD38(high) transitional human B cells reveals different regulatory profiles [J]. J Allergy Clin Immunol, 2016,137(5): 1577-1584 e1510. doi: 10.1016/j.jaci.2015.09.014

[7] Varghese JF, Kaskow BJ, von Glehn F, et al. Human regulatory memory B cells defined by expression of TIM-1 and TIGIT are dysfunctional in multiple sclerosis [J]. Front Immunol, 2024,15: 1360219. doi: 10.3389/fimmu.2024.1360219

[8] Lindner S, Dahlke K, Sontheimer K, et al. Interleukin 21-Induced Granzyme B-Expressing B Cells Infiltrate Tumors and Regulate T Cells [J]. Cancer Research, 2013,73(8): 2468-2479. doi: 10.1158/0008-5472.Can-12-3450

[9] Zhu H, Xu J, Wang W, et al. Intratumoral CD38(+)CD19(+)B cells associate with poor clinical outcomes and immunosuppression in patients with pancreatic ductal adenocarcinoma [J]. EBioMedicine, 2024,103: 105098. doi: 10.1016/j.ebiom.2024.105098

[10] Li H, Li XL, Cao S, et al. Decreased granzyme B(+)CD19(+)B cells are associated with tumor progression following liver transplantation [J]. Am J Cancer Res, 2021,11(9): 4485-4499. doi:

[11] Evans JG, Chavez-Rueda KA, Eddaoudi A, et al. Novel suppressive function of transitional 2 B cells in experimental arthritis [J]. J Immunol, 2007,178(12): 7868-7878. doi: 10.4049/jimmunol.178.12.7868

[12] Rosser EC, Oleinika K, Tonon S, et al. Regulatory B cells are induced by gut microbiota-driven interleukin-1beta and interleukin-6 production [J]. Nat Med, 2014,20(11): 1334-1339. doi: 10.1038/nm.3680

[13] Amu S, Saunders SP, Kronenberg M, et al. Regulatory B cells prevent and reverse allergic airway inflammation via FoxP3-positive T regulatory cells in a murine model [J]. J Allergy Clin Immunol, 2010,125(5): 1114-1124 e1118. doi: 10.1016/j.jaci.2010.01.018

[14] Iwata Y, Matsushita T, Horikawa M, et al. Characterization of a rare IL-10-competent B-cell subset in humans that parallels mouse regulatory B10 cells [J]. Blood, 2011,117(2): 530-541. doi: 10.1182/blood-2010-07-294249

[15] Wang XY, Wei Y, Hu B, et al. c-Myc-driven glycolysis polarizes functional regulatory B cells that trigger pathogenic inflammatory responses [J]. Signal Transduct Target Ther, 2022,7(1): 105. doi: 10.1038/s41392-022-00948-6

[16] Pati S, Mukherjee S, Dutta S, et al. Tumor-Associated CD19+CD39- B Regulatory Cells Deregulate Class-Switch Recombination to Suppress Antibody Responses [J]. Cancer Immunol Res, 2023,11(3): 364-380. doi: 10.1158/2326-6066.CIR-21-1073

[17] Yoshizaki A, Miyagaki T, DiLillo DJ, et al. Regulatory B cells control T-cell autoimmunity through IL-21-dependent cognate interactions [J]. Nature, 2012,491(7423): 264-+. doi: 10.1038/nature11501

[18] Yanaba K, Yoshizaki A, Asano Y, et al. IL-10-Producing Regulatory B10 Cells Inhibit Intestinal Injury in a Mouse Model [J]. American Journal of Pathology, 2011,178(2): 735-743. doi: 10.1016/j.ajpath.2010.10.022

[19] Yang M, Deng J, Liu Y, et al. IL-10-Producing Regulatory B10 Cells Ameliorate Collagen-Induced Arthritis via Suppressing Th17 Cell Generation [J]. American Journal of Pathology, 2012,180(6): 2375-2385. doi: 10.1016/j.ajpath.2012.03.010

[20] Lenert P, Brummel R, Field EH, et al. TLR-9 activation of marginal zone B cells in lupus mice regulates immunity through increased IL-10 production [J]. Journal of Clinical Immunology, 2005,25(1): 29-40. doi: 10.1007/s10875-005-0355-6

[21] Gray M, Miles K, Salter D, et al. Apoptotic cells protect mice from autoimmune inflammation by the induction of regulatory B cells [J]. Proceedings of the National Academy of Sciences of the United States of America, 2007,104(35): 14080-14085. doi: 10.1073/pnas.0700326104

[22] Ding Q, Yeung M, Camirand G, et al. Regulatory B cells are identified by expression of TIM-1 and can be induced through TIM-1 ligation to promote tolerance in mice [J]. Journal of Clinical Investigation, 2011,121(9): 3645-3656. doi: 10.1172/Jci46274

[23] Shalapour S, Font-Burgada J, Di Caro G, et al. Immunosuppressive plasma cells impede T-cell-dependent immunogenic chemotherapy [J]. Nature, 2015,521(7550): 94-U235. doi: 10.1038/nature14395

[24] Neves P, Lampropoulou V, Calderon-Gomez E, et al. Signaling via the MyD88 adaptor protein in B cells suppresses protective immunity during Salmonella typhimurium infection [J]. Immunity, 2010,33(5): 777-790. doi: 10.1016/j.immuni.2010.10.016

[25] Shen P, Roch T, Lampropoulou V, et al. IL-35-producing B cells are critical regulators of immunity during autoimmune and infectious diseases [J]. Nature, 2014,507(7492): 366-370. doi: 10.1038/nature12979

[26] Lino AC, Dang VD, Lampropoulou V, et al. LAG-3 Inhibitory Receptor Expression Identifies Immunosuppressive Natural Regulatory Plasma Cells [J]. Immunity, 2018,49(1): 120-+. doi: 10.1016/j.immuni.2018.06.007

[27] Matsumoto M, Baba A, Yokota T, et al. Interleukin-10-producing plasmablasts exert regulatory function in autoimmune inflammation [J]. Immunity, 2014,41(6): 1040-1051. doi: 10.1016/j.immuni.2014.10.016

[28] Zhang MX, Zeng GC, Yang QT, et al. Anti-tuberculosis treatment enhances the production of IL-22 through reducing the frequencies of regulatory B cell [J]. Tuberculosis, 2014,94(3): 238-244. doi: 10.1016/j.tube.2013.12.003

[29] Zhang M, Zheng X, Zhang J, et al. CD19(+)CD1d(+)CD5(+) B cell frequencies are increased in patients with tuberculosis and suppress Th17 responses [J]. Cell Immunol, 2012,274(1-2): 89-97. doi: 10.1016/j.cellimm.2012.01.007

[30] Choi JK, Yu CR, Bing SJ, et al. IL-27-producing B-1a cells suppress neuroinflammation and CNS autoimmune diseases [J]. Proc Natl Acad Sci U S A, 2021,118(47). doi: 10.1073/pnas.2109548118

[31] Zhang X, Deriaud E, Jiao X, et al. Type I interferons protect neonates from acute inflammation through interleukin 10-producing B cells [J]. J Exp Med, 2007,204(5): 1107-1118. doi: 10.1084/jem.20062013

[32] Brosseau C, Durand M, Colas L, et al. CD9(+) Regulatory B Cells Induce T Cell Apoptosis via IL-10 and Are Reduced in Severe Asthmatic Patients [J]. Front Immunol, 2018,9: 3034. doi: 10.3389/fimmu.2018.03034

[33] Yang G, Suo L, Hu S, et al. Characterization of the immune regulatory property of CD22(+) CD9(+) B cells [J]. Immunology, 2022,167(3): 328-339. doi: 10.1111/imm.13539

[34] Wang Z, Lu Z, Lin S, et al. Leucine-tRNA-synthase-2-expressing B cells contribute to colorectal cancer immunoevasion [J]. Immunity, 2022,55(6): 1067-1081 e1068. doi: 10.1016/j.immuni.2022.04.017

[35] Ibrahim EH, Aly MG, Opelz G, et al. Higher CD19+CD25(+) Bregs are independently associated with better graft function in renal transplant recipients [J]. BMC Nephrol, 2021,22(1): 180. doi: 10.1186/s12882-021-02374-2

[36] Kessel A, Haj T, Peri R, et al. Human CD19(+)CD25(high) B regulatory cells suppress proliferation of CD4(+) T cells and enhance Foxp3 and CTLA-4 expression in T-regulatory cells [J]. Autoimmun Rev, 2012,11(9): 670-677. doi: 10.1016/j.autrev.2011.11.018

[37] Han Y, Yu C, Yu Y, et al. CD25+ B cells produced IL-35 and alleviated local inflammation during experimental periodontitis [J]. Oral Dis, 2022,28(8): 2248-2257. doi: 10.1111/odi.13939

[38] Premkumar K, Shankar BS. TGF-betaR inhibitor SB431542 restores immune suppression induced by regulatory B-T cell axis and decreases tumour burden in murine fibrosarcoma [J]. Cancer Immunol Immunother, 2021,70(1): 153-168. doi: 10.1007/s00262-020-02666-w

[39] Huang H, Yao Y, Shen L, et al. CD24hiCD27+ Bregs within Metastatic Lymph Nodes Promote Multidrug Resistance in Breast Cancer [J]. Clin Cancer Res, 2023,29(24): 5227-5243. doi: 10.1158/1078-0432.CCR-23-1759

[40] Lundy SK, Fox DA. Reduced Fas ligand-expressing splenic CD5+ B lymphocytes in severe collagen-induced arthritis [J]. Arthritis Res Ther, 2009,11(4): R128. doi: 10.1186/ar2795

[41] Liu ZQ, Wu Y, Song JP, et al. Tolerogenic CX3CR1+ B cells suppress food allergy-induced intestinal inflammation in mice [J]. Allergy, 2013,68(10): 1241-1248. doi: 10.1111/all.12218
